# Pharmacy students’ attitudes and intentions of pursuing postgraduate studies and training in pharmacogenomics and personalised medicine

**DOI:** 10.1186/s40246-023-00474-8

**Published:** 2023-03-23

**Authors:** Dimitra Makrygianni, Margarita-Ioanna Koufaki, George P. Patrinos, Konstantinos Z. Vasileiou

**Affiliations:** 1 Laboratory of Pharmacogenomics and Individualized Therapy, Department of Pharmacy, University of Patras School of Health Sciences, University Campus, 26504 Patras, Greece; 2 Department of Genetics and Genomics, College of Medicine and Health Sciences, United Arab Emirates University, Al-Ain, Abu Dhabi United Arab Emirates; 3 Zayed Center for Health Sciences, United Arab Emirates University, Al-Ain, Abu Dhabi United Arab Emirates

**Keywords:** Pharmacy students, Attitudes, Intentions, Postgraduate studies, Training, Pharmacogenomics, Personalised medicine, Questionnaire survey

## Abstract

**Background:**

Pharmacists’ contribution to pharmacogenomics (PGx) implementation in clinical practice is vital, but a great proportion of them are not aware of PGx and its applications. This highlights the university education’s crucial role to prepare pharmacists to face future challenges in such a constantly evolving and demanding environment.

**Objectives:**

Our study aims to examine pharmacy students’ training satisfaction, knowledge, self-confidence and attitudes towards PGx on their intentions for postgraduate training in PGx and personalised medicine (PM).

**Methods:**

An initial model on students’ intention to pursue postgraduate training in PGx and PM and its predicting factors, based on the Theory of Planned Behaviour (TPB), was proposed. Based on it, a questionnaire was developed and distributed to 346 pharmacy students of all study years, capturing the selected factors influencing students’ intentions to postgraduate training in PGx and PM, as well as their demographics. Structural equation modelling (SEM) analysis was employed to determine the effects of both the examined factors and demographics on students’ intentions.

**Results:**

Students did not consider themselves adequately prepared for using PGx in clinical practice. Their attitudes towards PGx implementation were the most important factor influencing their intentions to pursue postgraduate training in PGx and PM. Other factors such as self-confidence and training satisfaction also affected students’ intentions, but to a lower extent. Students of the last two study years (40% of the whole sample) and male (36%) students stated to be less willing to pursue PGx-related studies in the future. Only 10% of the participants claimed to have undergone a recent PGx or genetic test, but this did not affect their intentions.

**Conclusion:**

There is an important gap in pharmacy school curriculum regarding PGx and PM training which coupled with the slow rate of PGx and PM implementation into clinical practice seems to restrain students’ aspiration to further expand their knowledge and horizons in terms of PGx and PM.

**Supplementary Information:**

The online version contains supplementary material available at 10.1186/s40246-023-00474-8.

## Introduction

Personalised medicine (PM) and especially pharmacogenomics (PGx), are gaining momentum worldwide. PM constitutes a state-of-the-art approach able to revolutionise the way current medicine works [1]. PGx is in the forefront of PM initiative, proposing an alternative treatment strategy in which individuals’ therapy scheme is tailored with respect of his/her genetic makeup. Currently, there are many clinical trials highlighting the clinical effectiveness of PGx in practice [1]. Indeed, it is demonstrated that PGx-guided treatment will reduce the incidence rate of adverse drug reactions (ADRs), decrease mortality and morbidity level along with hospital admissions, while it can increase drug efficacy by adjusting either drug dosage or switching to another medication [1]. Thanks to those advantages, overall disease and drug management can be improved to great extent. Apart from the clinical effectiveness of PGx, many studies conclude that PGx-guided treatment is a cost-effective strategy, and it will decrease healthcare expenditures. For all these points, PGx constitutes an important healthcare technology in drug management.

Pharmacists are the most specialised healthcare professionals in terms of drug management, and hence they play a pivotal role in the advancement and implementation of PM interventions in the clinical setting [2]. According to the latest American Society of Health System Pharmacists (ASHP) statements, pharmacists obtain the proper experience and education to interpret and apply PGx testing’s results [3]. In addition, based on Genetics/Genomics Competency Center (G2C2), it seems that pharmacists share distinct responsibilities in terms of PGx implementation and they should understand the ethnicity effect in genetic variance and subsequently in drug response and highlight the existence of PGx guidelines for different medications [4]. In other words, a pharmacist is authorised to adjust drug dosage, select a drug therapy, and monitor patients’ progress with regard to PGx results. Assisting other healthcare providers (i.e. physicians, nurses) in making clinical decision is also possible along with patient counselling related to their genetic results [3].

Even if pharmacists’ contribution to PGx clinical implementation is vital, they have a minimal involvement in the field. As reported in many studies, a great percentage of pharmacists are not aware of PGx and its applications; some of them do not know how pharmacists can be involved in the field and what are their responsibilities, while others claim to have limited or inadequate training [2, 5–9]. Low participation rate can decelerate the adoption of PM applications. According to Koufaki et al., 2021, education is the key to face new challenges in such a constantly evolving environment and to achieve better results in terms of PGx adoption rate and pharmacists’ response [10]. By initiating future generations of pharmacists to the principles and best practices of PGx at an early stage of their undergraduate education, it is possible to increase pharmacists’ self-confidence, disseminate new practices and thus enhance their participation in the sector [11, 12].

Admittedly, there are some studies that focus on understanding pharmacists’ and pharmacy students’ level of awareness, perceptions, attitudes and intentions about PGx applications in clinical practice. However, there is a significant gap in the literature regarding both pharmacists’ and pharmacy students’ intentions to continue their education in the field of PGx and PM [13–16]. This gap may pose a barrier in the widespread adoption of clinical PGx.

In this study, we aim to investigate the perceptions and opinions of undergraduate students of all years of studies from the Department of Pharmacy at the University of Patras in Greece. This department is the first pharmacy department in Greece that has a dedicated PGx module both in the undergraduate and graduate curriculum, towards some key aspects of PGx and PM. Using a questionnaire survey based on the Theory of Planned Behavior (TPB), we seek to evaluate the impact of selected factors and demographics on student’s intentions to pursue postgraduate training in PGx and PM.

## Materials and methods

### Research framework of the proposed model on students’ intention to pursue postgraduate training in PGx and PM and its predicting factors

Literature review revealed that there was a lack of publications focussing on the intentions, and its predicting factors, of health science students' to pursue PGx and PM-related training after graduation. The Theory of Planned Behaviour (TPB) has been continuously applied in a great spectrum of healthcare issues, including the investigation of health science students’ intentions for further education [17–20]. According to TPB, attitudes are considered as a critical factor affecting individuals’ intentions for future behaviour. Therefore, an extensive literature review was conducted regarding the attitudes, perceptions and intentions of health science students regarding PGx and PM. Prior research revealed that students’ attitudes towards PGx and PM application and adoption were related, among other factors, to their level of knowledge, their satisfaction from PM and PGx training and their self-confidence to apply PGx and PM in clinical practice [6, 7, 13–15, 21–23].

Literature review findings were discussed with pharmacy senior undergraduate and postgraduate (Master and PhD) students, as well as with experts in the field of PGx and PM. Experts were mainly coming from the Laboratory of PGx and Individualized Therapy, Department of Pharmacy, University of Patras, and had experience in research and survey development. Upon these discussions, the following factors were selected to probe their effect on the intention of pharmacy students to be further trained in the mentioned subjects/disciplines: 1) the self-confidence to apply PGx in clinical practice, 2) the satisfaction with the training regarding the application of PGx in clinical practice, 3) the prevailing attitudes-intentions for the application of PGx and PM and 4) the level of knowledge about PGx and PM.

**Fig. 1:**
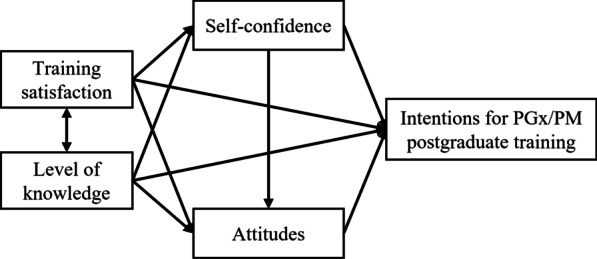
Research framework of students’ intention and its predicting factors to pursue postgraduate training in PGx and PM

The proposed research framework of students’ intention and its predicting factors to continue training in PGx and PM after graduation is presented in Fig. 1. Mahmutovic et al. (2018) concluded that pharmacy students whose curriculum contained a course in PGx and PM hold more positive attitudes and were more willing to continue their training in this topic compared to students without previous relevant training [14]. Moreover, Alzoubi et al. (2020) found that Jordanian medical students and physicians expressed rather positive attitudes towards PGx, and, at the same time, high interest in attending corresponding training sessions or workshops in the future [24]. Additionally, according to the pharmacogenomics/genomics literacy framework for pharmacists (PGLP) developed by Rahma et al. (2021) attitudes played a positive influential role on pharmacists’ intentions to continuing medical education [7]. Therefore, we assumed that attitudes influence positively students’ intentions to pursue postgraduate training in PGx and PM.

Rahma et al. (2021) framework also pinpointed that both pharmacists’ knowledge and skills, often assessed by self-confidence, were correlated with their attitudes, and altogether affected their intentions to undergo a PGx training in the future [7]. Grace et al. (2021) examined the impact of Personal Genomic educational testing (PGET) on student knowledge, comfort, and attitudes related to PGx in US students, and they noticed a simultaneous improvement in comfort with PGx clinical skills, PGx patient education, and attitudes towards PGx for all participants (receiving or not PGET) over the course of the study [22]. These findings are in line with the results of other studies mentioning that students’ PGx knowledge is positively correlated with their self-confidence (readiness) and attitudes towards PGx implementation in clinical practice [13, 15]. Thus, we hypothesised that higher levels of self-confidence in PGx implementation in practice will be positively associated with favourable attitudes towards PGx and with greater intentions for PGx and PM postgraduate training. Accordingly, higher levels of PGx knowledge will be positively associated with higher self-confidence in PGx implementation, more favourable attitudes towards PGx and with greater intentions for PGx and PM postgraduate training.

Both Mahmutovic et al. (2018) and Cheung et al. (2021) studies highlighted that the more students considered their study curriculum as well-designed for PGx understanding, the more they expressed positive attitudes towards PGx and were interested to get involved in this area of research or professional practice [14, 21]. Considering the above, we assumed that higher levels of training satisfaction will be positively associated with higher self-confidence in PGx implementation, more positive attitudes towards PGx and with greater intentions for PGx and PM postgraduate training.

### Survey development

Survey’s tool development involved a multistage process. Initially, we combined relevant questionnaire surveys [13–15, 21, 23, 25–28] and the aforementioned discussions as the basis to develop questionnaire’s items associated with students’ intentions for postgraduate studies in PGx and PM and to determine the factors included in the proposed research model. At this stage, we decided to include another statement related to students’ evaluation of teaching methods used in PGx and PM education to facilitate the interpretation of the survey results. Next, the content validity of the selected items was examined by experts in the field to ascertain that all crucial items of each factor/construct were included. Draft questionnaire was then pretested through cognitive interviews with 25 pharmacy students of all years from the University of Patras to evaluate its clarity, content, length, and measurement scale [29]. The final version of questionnaire was reviewed mainly about the wording and clarity of some items.

It consisted of 38 questions divided into 7 different sections: (1) evaluation of teaching tools used for the PGx and PM training, (2) self-confidence to apply PGx in clinical practice, (3) satisfaction with the training regarding the application of PGx in clinical practice, (4) prevailing attitudes-intentions for the application of PGx and PM, (5), intentions for postgraduate training in PGx and PM, (6) level of knowledge about PGx and PM and (7) student demographics (such as gender, year of study, possession of another BSc degree, and if they or a family member are under chronic medication and have undergone a recent PGx or genetic test) (see Additional file 2). All main sources considered, along with the measurement scale used to develop the survey, are demonstrated in Table 1. Moreover, an introductory cover page was attached in the beginning of the questionnaire to explain study’s purpose and objectives and to provide instructions on its completion. Additionally, definitions of PM and PGx were provided, and it was assured that participants anonymity and confidentiality would be preserved. The survey questionnaire required around 10 min to complete.

**Table 1:** Sources and scales of measurement for each questionnaire section

Factor	Sources	Number of items	Scale
Evaluation of teaching tools	[23]	7	1–7: not at all useful (1)–extremely useful (7)
Self-confidence in implementing PGx	[13, 22, 25, 28]	5	1–7: not at all confident (1)–extremely confident (7)
Satisfaction with PGx training	[21]	3	1–7: strongly disagree (1)–strongly agree (7)
Attitudes-Intentions for PGx implementation	[14, 15, 21, 22]	6	1–7: strongly disagree (1)–strongly agree (7)
Intentions for postgraduate training in PGx and PM	[7, 14, 21]	2	1–7: strongly disagree (1)–strongly agree (7)
Level of knowledge in PGx and PM	[15, 25, 27]	10	Agree, disagree, Do not know

### Study sample

Study sample consisted of 346 undergraduate students from the Department of Pharmacy of the University of Patras, Greece. Students of all academic years were asked to complete the questionnaire before or after their lectures during April (spring semester) 2022. Around two-thirds (64.5%) of the participants were women in line with the gender distribution of the Department’s student population (Table 2). Fifth-year students (27.7%) were the most willing to participate in the survey, closely followed by the third year (24.3%), while fourth year (12.4%) were the least disposed to fill in the questionnaire. Notably, PGx principles and applications are taught in the course “Molecular Genetics and PGx” in the spring semester of the second study year. PGx-related material is also briefly covered in other modules within pharmacy curriculum. Moreover, 9% of the students stated they hold another BSc degree. Only 10% of the participants claimed that they or a relative of theirs have undergone a recent PGx or genetic test, while almost 67% stated that they or a family member was under chronic medication.

**Table 2:** Sample descriptive statistics (valid %; *N* = 346)

Variables		*N*	%	Variables		*N*	%
Gender	Male	123	35.5	Recent PGx or genetic test	Yes	33	9.5
	Female	223	64.5		No	313	90.5
Study Year	1st	62	17.9	Chronic medication	Yes	215	62.1
	2nd	61	17.6		No	131	37.9
	3rd	84	24.3	Holder of another BSc degree	Yes	32	9.2
	4th	43	12.4		No	314	90.8
	5th	96	27.8				

### Data analysis

The research data were analysed by the SPSS and AMOS statistical programmes (both versions 28; IBM, NY, USA). Data analysis included frequencies and percentage of valid responses (valid %) and descriptive statistics (mean value, standard deviation (Std.Dev)). Mann–Whitney and Kruskal–Wallis tests were applied to identify the scale of differences among student groups based on their demographics (i.e. gender, study year, another BSc degree, PGx/genetic test, chronic medication). There is a debate whether parametric (t test and ANOVA) or nonparametric (Mann–Whitney and Kruskal–Wallis) tests should be used for group comparisons of item data selected with Likert scale. However, relevant studies concluded that both procedures, generally, have similar power to detect differences among groups [30, 31]. In our study, nonparametric tests were preferred, as the Kolmogorov–Smirnov test results indicated that the survey data were not normally distributed for almost all items.

Structural equation modelling (SEM) analysis (IBM AMOS: generalised least squares (GLS) method for parameter estimation, regression coefficients estimation and statistical fits of the structural model evaluation) was utilised to discern the direct, indirect and total effects of both the examined factors and demographics on students’ intentions to pursue postgraduate training in PGx and PM [32]. A direct effect refers to the direct impact of an independent (exogenous) variable on a dependent (endogenous) variable, an indirect effect is the effect of an independent variable on a dependent variable that goes through a mediator (the independent variable influences the mediator which then affects the dependent variable) and a total effect is the sum of the direct and indirect effects of an independent on a dependent variable [33]. Prior to the structural model analysis, a measurement model analysis was conducted to examine the relationship between the latent variables and their measures, consisting of model fit statistics (CMIN/DF, CFI, TLI, NFI, IFI, RFI, RMSEA, SRMR) and the factor loadings of each factor [33]. Moreover, Cronbach’s alpha and composite reliability (CR) test were used to measure the examined factors’ construct reliability. Average variance extracted (AVE) for each construct was also calculated to assess convergent validity. Discriminant validity was assessed by the heterotrait-monotrait ratio of correlations (HTMT) method. Additionally, the possible influence of common method variance (CMV) was measured by Harman’s single factor test [33].

## Results

### Students’ perceptions on the examined factors affecting their intentions for postgraduate training in PGx and PM

**Fig. 2:**
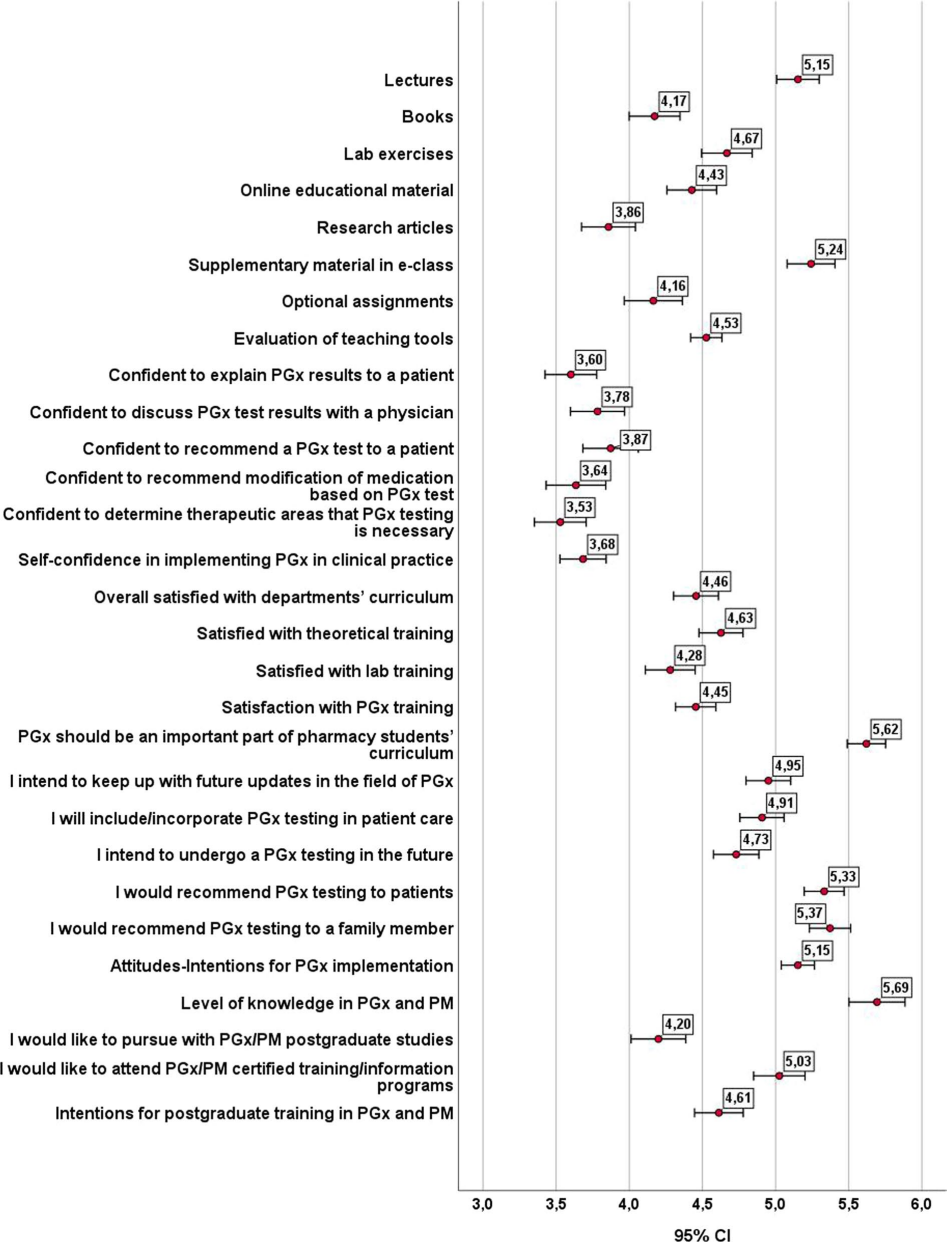
Descriptive statistics (mean and standard error of means) of student answers

Students assessed the teaching tools used in PGx modules to be moderately to rather useful (Fig. 2 and Table 3). Lectures and supplementary material in e-class were perceived as the most useful tools (mean around 5.2), followed by laboratory training (mean 4.7). Books and formative assignments were considered as moderately helpful (mean around 4.2). Study year was the only demographic variable to influence students’ evaluation of lectures, laboratory training, e-class material and optional assignments (Additional file 1: Tables S1–S5). Indeed, second-year students were indicated to be the most pleased with available teaching tools in contrast to fifth-year students. Second-year students were very satisfied with lectures, laboratory training and e-class material (means from 5.3 to 5.9), while third-year students found lectures and e-class material quite valuable (means around 5.6).

**Table 3:** Frequencies (valid %) of student answers

	Variables/factors	1	2	3	4	5	6	7
TCH1	Lectures	2.0	1.4	8.4	17.1	26.3	28.0	16.8
TCH2	Books	7.2	8.7	18.2	23.4	18.2	16.5	7.8
TCH3	Laboratory exercises	5.2	5.5	10.7	24.6	17.9	22.3	13.9
TCH4	Online educational material	4.9	8.1	13.0	27.2	16.5	20.8	9.5
TCH5	Research articles	11.6	13.6	15.9	22.0	17.1	13.3	6.6
TCH6	Supplementary material in e-class	3.8	2.6	7.5	11.8	22.8	28.9	22.5
TCH7	Optional assignments	13.0	8.7	12.4	20.5	18.5	13.9	13.0
*Evaluation of teaching tools*								
CNF1	Confident to explain PGx results to a patient	12.1	16.5	20.2	21.4	14.5	10.7	4.6
CNF2	Confident to discuss PGx test results with a physician	11.6	17.1	14.7	19.4	18.5	13.0	5.8
CNF3	Confident to recommend a PGx test to a patient	13.0	11.8	16.8	19.7	19.1	11.3	8.4
CNF4	Confident to recommend modification of medication based on PGx test	19.4	14.2	14.7	16.5	13.6	13.9	7.8
CNF5	Confident to determine therapeutic areas that PGx testing is necessary	14.7	15.9	17.6	22.5	15.0	11.0	3.2
*Self-confidence in implementing PGx in clinical practice*								
STF1	Satisfied with theoretical training	3.2	4.3	8.4	32.4	24.9	16.2	10.7
STF2	Satisfied with laboratory training	7.2	8.4	11.3	28.0	19.4	18.8	6.9
STF3	Overall satisfied with departments’ curriculum	3.2	6.9	12.4	28.6	23.4	18.2	7.2
*Satisfaction with PGx training*								
ATT1	PGx should be an important part of pharmacy students’ curriculum	0.3	1.2	2.6	16.2	22.8	25.7	31.2
ATT2	I intend to keep up with future updates in the field of PGx	2.3	3.5	6.9	25.4	24.3	21.1	16.5
ATT3	I will include/incorporate PGx testing in patient care	3.5	2.9	5.2	25.4	27.7	21.4	13.9
ATT4	I intend to undergo a PGx testing in the future	3.2	5.2	6.4	29.5	25.4	17.1	13.3
ATT5	I would recommend PGx testing to patients	1.4	1.4	4.0	17.3	26.3	30.1	19.4
ATT6	I would recommend PGx testing to a family member	0.9	3.2	3.2	17.1	24.6	28.6	22.5
*Attitudes-Intentions for PGx implementation*								
INT1	I would like to pursue with postgraduate studies related to PGx and PM	8.4	13.6	8.1	26.3	19.1	12.4	12.1
INT2	I would like to attend certified training or information programmes related to PGx and PM	4.6	4.9	7.5	16.8	19.9	24.9	21.4
*Intentions for postgraduate training in PGx and PM*								

Students’ satisfaction related to training received in PGx clinical implementation is in line with their evaluation of teaching tools. They appear to be moderately to rather satisfied with theoretical and laboratory training along with department’s curriculum, as 45% to 50% of participants agreed with the relevant statements and means ranged from 4.3 to 4.6 (Fig. 2 and Table 3). Again, study year was the only demographic variable affecting students’ views (Additional file 1: Tables S1–S5), with second-year students being the most satisfied (mean 5.3) and fifth-year the least (mean 4.0). Third-year students were rather satisfied with their theoretical training (mean 4.8), but moderately with their laboratory training (mean 3.7), which is attributed mainly to the special circumstances that laboratory exercises were conducted during the last two years because of COVID-19 restrictions. Students claimed to be moderately confident in implementing PGx in clinical practice, as the means of all relevant statements ranged from 3.5 to 3.9 (Fig. 2 and Table 3). Study year only affected students’ self-confidence, mainly due to significant divergences between first and second-year students (Additional file 1: Tables S1–S5).

Students shared very positive attitudes towards PGx and its implementation in clinical practice. Around 80% agreed that PGx should be an important part of pharmacy students’ curriculum and they would recommend PGx testing to patients or a family member (Fig. 2 and Table 3). Moreover, almost 60% agreed that they intended to keep up with future updates in the field of PGx and incorporate PGx testing in patient care. Female students were more positive than their male classmates concerning the significance of PGx in their curriculum, while female students intended to be updated about future advances in the field and to integrate PGx in patient care. Students (or a family member of theirs) receiving chronic medication were more inclined to recommend PGx testing to patients or a family member, whereas those holding another BSc degree were less willing to undergo a PGx testing in the future. First-year, closely followed by second-year, students held more positive attitudes and were more disposed to adopt PGx in clinical practice and keep up with future updates.

**Fig. 3:**
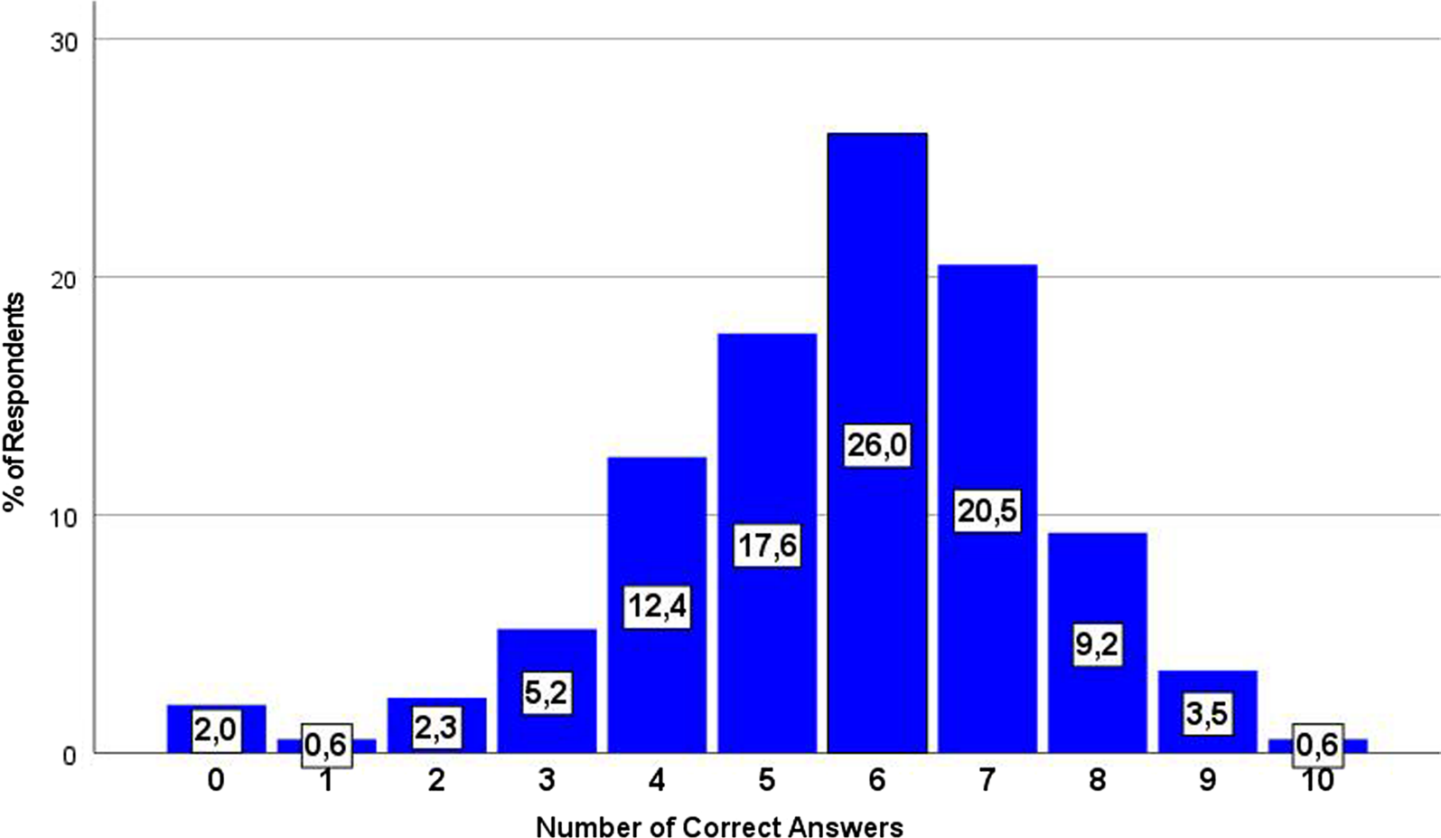
Students’ overall level of knowledge in PGx and PM (Valid %, *N* = 346)

**Fig. 4:**
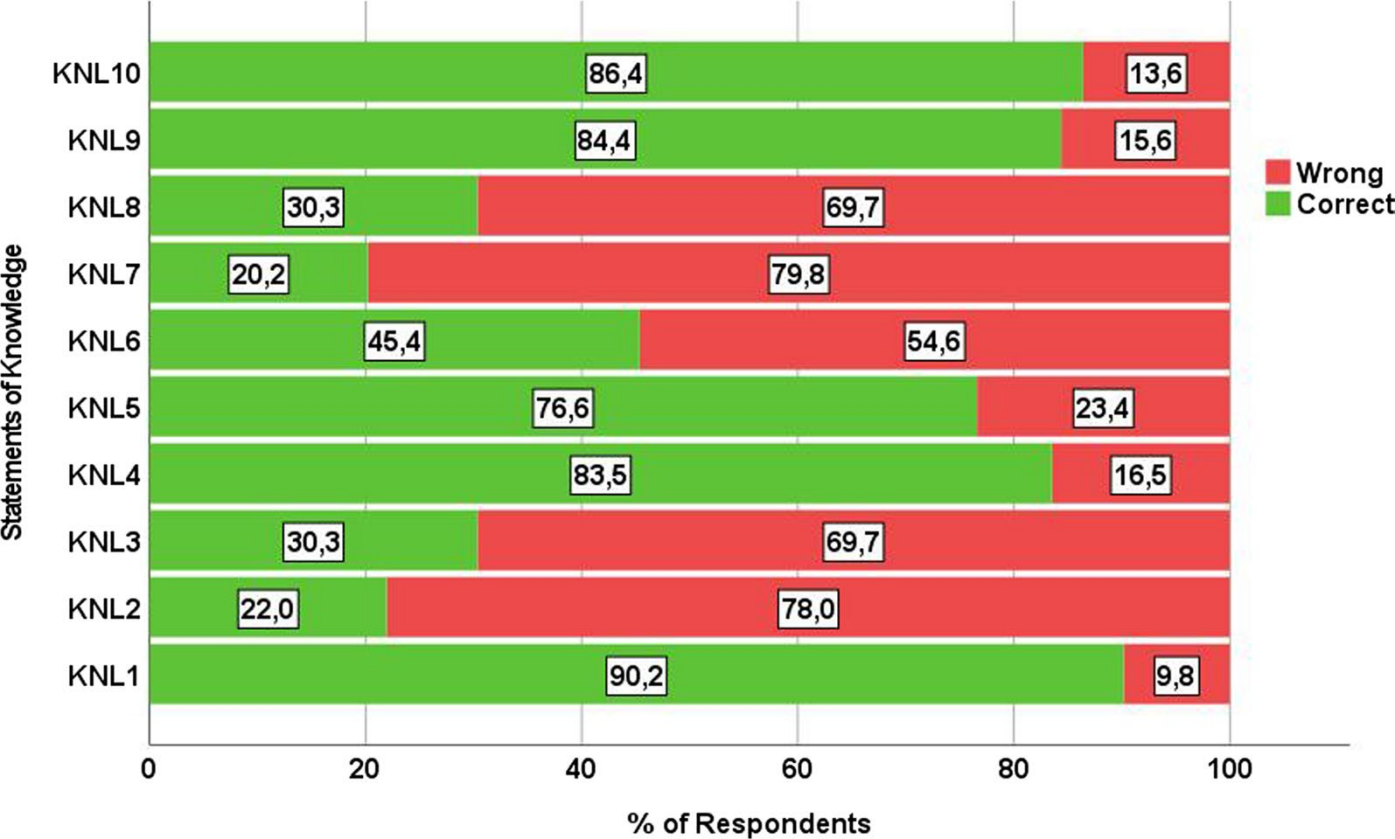
Percentage of students answering correctly and wrongly to each statement of knowledge about PGx and PM (Valid %, *N* = 346)

Individuals’ level of knowledge on PGx and PM was measured by an index, (0 to 10), concerning the total number of correct answers given at the 10 relevant statements (Fig. 3). Students’ level of knowledge in PGx and PM was found moderately satisfactory (Figs. 2, 3 and 4). Approximately 75% of them gave 4 to 7 correct answers, whereas only 4% of the respondents got a score of 9 out of 10 (mean = 5.7, median = mode = 6). Students (or a family member of theirs) receiving chronic medication scored on average 0.5 units higher than others. Moreover, students of the last three study years presented an almost equal level of knowledge in PGx and PM, but much higher than those of the first and second year (Additional file 1: Tables S1–S5).

Students claimed to be moderately willing to pursue postgraduate studies related to PGx and PM, Almost 45% of them agreed with the pertinent clause and one-quarter of them were neutral (mean 4.2). However, they were quite disposed to attend relevant certified training or information programmes, since about two-thirds of them agreed with the relevant statement and only 17% disagreed (mean 5.03) (Fig. 2 and Table 3). Female students were more inclined to improve their knowledge and skills on PGx and PM, while those possessing another BSc degree stated a lower interest. Students’ interest for postgraduate training in PGx and PM declined as they ascended the study year. Thus, while students of the first 3 years appeared quite willing for postgraduate training, fourth- and, especially, fifth-year students were, to some extent, reluctant, particularly to attend a relevant MSc course (Additional file 1: Tables S1–S5).

### SEM analysis of key determinants on student intentions for postgraduate training in PGx and PM

The measurement model of the four factors (Training Satisfaction, Self-Confidence, Attitudes and Intentions) was tested by Confirmatory Factor Analysis (CFA). The level of knowledge on PGx and PM was measured by an index, of a scale from 0 to 10; therefore, it was not considered in the measurement model analysis. All values of the model-fit measures calculated to assess the model’s overall goodness of fit were within the corresponding acceptance levels. Specifically, the values computed by AMOS were: CMIN/DF = 1.512 < 3–5, CFI = 0.986 ≥ 0.90, TLI = 0.981 ≥ 0.90, NFI = 0.960 ≥ 0.90, IFI = 0.986 ≥ 0.90, RFI = 0.945 ≥ 0.90, RMSEA = 0.39 (0.023 – 0.052) < 0.08 and SRMR = 0.041 < 0.08 (Collier 2020; Kline, 2016). According to Table 4, the factor loadings of each item on the respective constructs are well above the suggested threshold of 0.5; therefore, all items were eligible for the next stages of analysis. However, ATT6 item (*I would recommend pharmacogenomic testing to a family member*) was removed from the analysis because of its high correlation with ATT5 item (0.848).

**Table 4:** Factor loadings, reliability and convergent validity of measurement model

Constructs-items	Loadings	Cronbach’s α	CR	AVE
*Self-confidence*		0.901	0.859	0.553
CNF1	0.873			
CNF2	0.827			
CNF3	0.713			
CNF4	0.647			
CNF5	0.626			
*Training satisfaction*		0.841	0.846	0.648
STF1	0.747			
STF2	0.749			
STF3	0.909			
*Attitudes*		0.849	0.851	0.536
ATT1	0.705			
ATT2	0.876			
ATT3	0.771			
ATT4	0.637			
ATT5	0.646			
*Intentions*		0.810	0.810	0.681
INT1	0.815			
INT2	0.835			

Cronbach’s alpha and composite reliability (CR) verified the construct reliability of each model construct (Table 4). All Cronbach’s alpha values were quite higher than the acceptance level of 0.70 [34], and CRs, ranging from 0.81 to 0.86, were well above the threshold value of 0.70 [35]. The average variance extracted (AVE) for all constructs was found higher than the benchmark of 0.50 (Table 4), indicating the convergent validity of the factors included in model [36]. The discriminant validity was confirmed by the heterotrait-monotrait ratio of correlations (HTMT) method, as all ratios ranged between 0.202 and 0.797 (Table 5), below the suggested critical value of 0.85 [37]. Finally, in our measurement model the Harman’s single factor test showed that the total variance for a single factor was 36.77%, considerably lower than the recommended threshold of 50%, and hence, we assumed that the common method bias did not constitute a significant threat to the validity of our research findings [38, 39].

**Table 5:** HTMT ratios for discriminant validity

	Self-Confidence	Training Satisfaction	Attitudes
Training Satisfaction	0.202		
Attitudes	0.397	0.357	
Intentions	0.266	0.305	0.797

Mann–Whitney and Kruskal–Wallis tests revealed significant differences on some of students’ answers according to gender, study year, possession of another BSc degree and receiving chronic medication. Thus, these demographics were included in SEM analysis as control variables. A binary variable was used for “study year” (1st–3rd: 0; 4th–5th: 1) to simplify the SEM analysis. Third-year students were grouped together with their first- and second-year classmates, as they expressed relatively similar intentions for postgraduate training in PGx and PM. This may be explained by the fact that fourth and fifth-year students are approaching to the completion of their studies, and consequently they are more settled regarding their intentions for postgraduate training including PGx and PM.

**Fig. 5:**
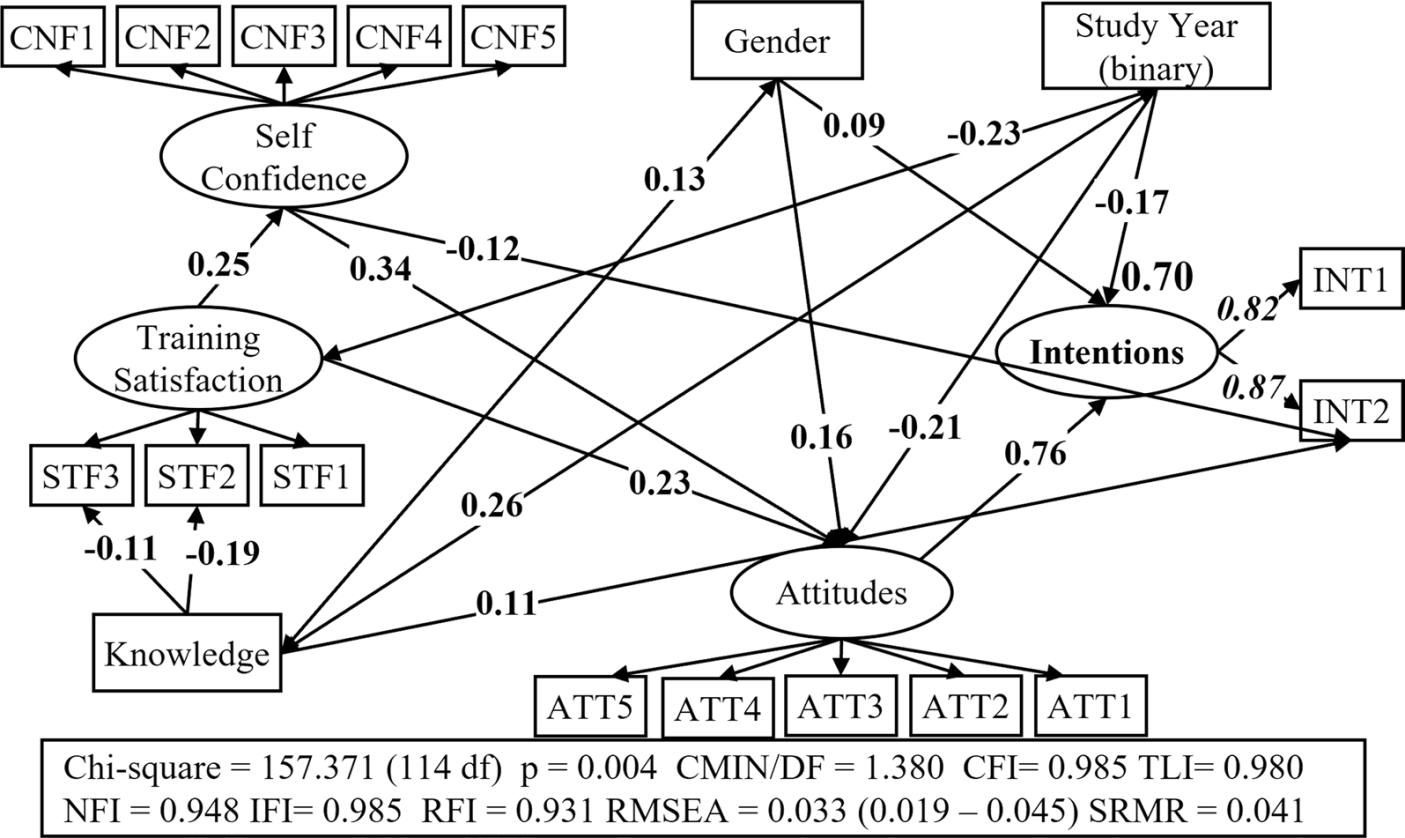
Synoptic SEM diagram of the factors influencing students’ intention to pursue postgraduate training in PGx and PM

Figure 5 outlines the statistically significant effects of key determinants on student intentions. More details are presented in Figure S1 and Tables 6 and 7. The values of the global fit statistics (CMIN/DF < 2–5, CFI ≥ 0.90, TLI ≥ 0.90, NFI ≥ 0.90, IFI ≥ 0.90, RFI ≥ 0.90, RMSEA < 0.08, SRMR < 0.08) indicated a good model fit [32, 33]. Moreover, the coefficient of determination (*R*^2^) was 0.70 signifying, as well, a good model fit. Attitudes towards PGx implementation were the only factor which directly influenced students’ intentions to pursue postgraduate training in PGx and PM. The high value of the pertinent standardised regression weight, 0.76, indicated a very strong positive impact of attitudes on intentions for postgraduate training.

**Table 6:** Regression weights and covariances for the SEM model

Regression weights			Standardised estimate	S.E.	C.R.	*P*
Intentions	** ← **	Attitudes	0.755	0.11	11.303	***
Attitudes	** ← **	Self_Confidence	0.347	0.047	5.686	***
Attitudes	** ← **	Training_Satisfaction	0.227	0.049	3.823	***
Self_Confidence	** ← **	Training_Satisfaction	0.249	0.068	3.903	***
INT2	** ← **	Self_Confidence	−0.109	0.063	−2.486	0.013
INT2	** ← **	Knowledge	0.107	0.034	2.893	0.004
STF2	** ← **	Knowledge	−0.189	0.038	−4.383	***
STF3	** ← **	Knowledge	−0.112	0.031	−2.908	0.004
Intentions	** ← **	Year_binary	−0.172	0.134	−3.805	***
Attitudes	** ← **	Year_binary	−0.208	0.094	−3.972	***
Training_Satisfaction	** ← **	Year_binary	−0.228	0.127	−3.915	***
Intentions	** ← **	Gender	0.158	0.129	2.214	0.027
Attitudes	** ← **	Gender	0.094	0.093	3.131	0.002

Covariances			Correlations	S.E.	C.R.	*p*
Knowledge	** ↔ **	Year_binary	0.263	0.049	4.761	***
Knowledge	** ↔ **	Gender	0.126	0.045	2.408	0.016

***p < 0.001

**Table 7:** Standardised direct, indirect and total effects for the SEM model

	Attitudes	Self_Confidence	Training_Satisfaction	Knowledge	Year_binary	Gender
*Total*						
Intentions	0.755	0.262	0.236	0.000	−0.383	0.214
Attitudes	0.000	0.347	0.313	0.000	−0.280	0.158
Self_Confidence	0.000	0.000	0.249	0.000	−0.057	0.000
Training_Satisfaction	0.000	0.000	0.000	0.000	−0.228	0.000
INT1	0.621	0.215	0.194	0.000	−0.315	0.176
INT2	0.657	0.119	0.179	0.107	−0.328	0.186
*Direct*						
Intentions	0.755	0.000	0.000	0.000	−0.172	0.094
Attitudes	0.000	0.347	0.227	0.000	−0.208	0.158
Self_Confidence	0.000	0.000	0.249	0.000	0.000	0.000
Training_Satisfaction	0.000	0.000	0.000	0.000	−0.228	0.000
INT1	0.000	0.000	0.000	0.000	0.000	0.000
INT2	0.000	−0.109	0.000	0.107	0.000	0.000
*Indirect*						
Intentions	0.000	0.262	0.236	0.000	−0.211	0.120
Attitudes	0.000	0.000	0.086	0.000	−0.071	0.000
Self_Confidence	0.000	0.000	0.000	0.000	−0.057	0.000
Training_Satisfaction	0.000	0.000	0.000	0.000	0.000	0.000
INT1	0.621	0.215	0.194	0.000	−0.315	0.176
INT2	0.657	0.228	0.179	0.000	−0.328	0.186

Self-confidence and training satisfaction affected students’ intentions only indirectly, as they exhibited a moderate positive influence on attitudes (total effects: 0.35 and 0.31, respectively). Furthermore, training satisfaction exerted a relatively low (0.25) positive effect on self-confidence. However, self-confidence was found to have a rather low negative (−0.11), but still significant, impact solely on students’ intentions to attend certified training or information programmes related to PGx and PM (INT2). Therefore, the positive total, and indirect, effects of both self-confidence and training satisfaction on intentions for postgraduate training were relatively low: 0.26 and 0.24, respectively.

The level of knowledge was the only examined factor of the proposed research model that had neither direct nor indirect impact on students’ intentions for postgraduate training, in general. However, it appeared to have a quite low positive impact (0.11) on students’ intentions to attend certified training or information programmes related to PGx and PM (INT2). Although no significant relationship was established between the level of knowledge and the training satisfaction construct, a low negative relationship was found between the level of knowledge and the satisfaction with both the laboratory training (STF2) and the departments’ curriculum (STF3) (−0.19 and −0.11, respectively).

Concerning control variables, study year, primarily, and gender, to a less degree, influenced students’ intentions for postgraduate training both directly and indirectly. Specifically, in line with the Kruskal–Wallis test results, students of the last two study years appeared to be considerably less willing to pursue postgraduate training in PGx and PM, less zealous to implement PGx and PM in clinical practice and less satisfied with their training than their classmates of the first 3 years. Female students were more disposed to pursue postgraduate training and hold more favourable attitudes towards PGx and PM than their male classmates.

## Discussion

To the best of our knowledge, this is the first study to examine the effects of selected factors (e.g. attitudes, knowledge, self-confidence, training satisfaction) on pharmacy undergraduate students’ intentions to pursue PGx-related studies in the future using TPB.

Our study showed that the upcoming generations of professional pharmacists were aspired by the advantages of PGx and shared a positive attitude towards its integration in clinical practice. In parallel, it was observed that they were willing to pursue postgraduate studies in this field, despite the fact that they expressed a moderate satisfaction from their department’s curriculum, and they did not feel well-prepared to clinically implement PGx. Furthermore, SEM analysis’ results indicated a good fit of the proposed model, pinpointing that some of the examined factors such as attitudes, training satisfaction and self-confidence exerted a significant impact on students’ intentions to pursue further education in PGx and PM field. Results from SEM analysis also demonstrated that attitudes were the most influential factor, directly affecting respondents’ intention to continue their studies in the sector, a fact that is congruent with what Koufaki et al., 2022 showed in a study among Malaysian and Greek healthcare students [40].

Students mentioned that lectures, supplementary material and laboratory training were the most useful educational tools. This tendency is observed across all academic years. Second-year students claimed that lectures were the most valuable education tool. PGx- and PM-dedicated module takes place at spring semester of second year as in 21% of pharmacy schools in the USA [41] and therefore corresponding students were affected by it.

As far as the self-confidence concerns, most of the respondents did not consider themselves as adequately prepared to apply PGx in clinical practice except of first-year students. It is likely that first-year students overestimated their competencies and readiness in PGx implementation. Second-year students were the least self-confident probably because they realised their limited knowledge on PGx and PM subjects, while the students of the last three study years were, slightly more self-confident. In other studies individuals also characterised their self-confidence in clinically applying PGx as weak [26, 42]. Moreover, according to de Denus et al., 2013, only a 7.7% of Canadian pharmacists claimed to feel comfortable to integrate PGx in their daily practice while only a quarter of USA pharmacists stated to be able to interpret PGx results to their patients’ treatment [43, 44]. Moreover, Bank et al., 2018 found that an almost 28% of pharmacy students thought that they were adequately trained and prepared to interpret PGx testing data and change medication or dosage [26].

Moreover, participants characterised their curriculum’s satisfaction as intermediate, and they weren’t very satisfied. Final-year students shared the lowest level of satisfaction regarding their studies which may be attributed, inter alia, to their internships at community and hospital pharmacies. These results are consistent with other studies in which 60.3% of individuals believed that their university education was insufficient and only a third of them supposed that lectures were comprehensive and clear [8, 15]. This descending trend highlighted the insufficiency of PGx-related material in latent modules and the students’ demand for a more tailored educational scheme that focuses more on learner’s skills, needs and wants. Admittedly, new pedagogies have incorporated evidence-based learning as an alternative teaching approach with the objective to better prepare students for their future professional career [7, 45].

Furthermore, participants from all academic years expressed a very positive attitude towards PGx implementation in clinical practice, a strong indication of being aware and convinced of PGx benefits. Students’ positive attitude was also highlighted by their willingness to undergo a PGx testing or to recommend it to patients or friends. Our results are in line with another study occurred in Greece by Siamoglou et al., 2021 in which the majority of health students had a positive opinion about the topic and they mentioned that they would perform a genetic test in the future. Other studies agreed with our findings as well and pinpointed that positive attitude was associated with a person’s intention to adopt a relevant clinical intervention [14, 40, 46].

Positive attitude is also translated into positive intention, as it was hypothesised in our proposed model. Students’ majority were prone to pursue postgraduate studies in the future or continue getting updated about the newest advances in the field. This may be also attributed to the fact that in Greece, all faculties–departments of medicine and pharmacy include courses focussing on PGx and PM in their undergraduate curricula, according to the information provided by their webpages. Additionally, there are three postgraduate programmes with a concentration on this field taught in the biggest Universities of Greece and the relevant academic research laboratories offer doctorate programmes, as well. These aspects highlight the high interest of Greek academics in promoting PGx and PM and raising students’ awareness about the importance of their clinical implementation.

Consistent with previous studies, this observation is important and encouraging. In particular, Mahmutovic et al., 2018 mentioned that 60% of the students asked would like to continue their studies in the field, Schwartz et al. 2017 demonstrated that 97% of the hospital pharmacists expressed interest in continuing their training, while in Indonesia, 94.2% of pharmacists were interested in future education [42] along with Jordan where a 75% of pharmacists expressed their will to participate in postgraduate training activities or seminars [14, 42, 44, 47]. Based on Pop et al., 2022, and Filiptsova et al., 2015, new generation of pharmacists were more progressive and willing to implement innovative technologies and interventions in their clinical practice with the objective to improve patient experience and provide them with better quality of services [8, 48]. To achieve it, it is fundamental to receive official training and accreditation. It is true that in many studies, respondents, especially, professionals pinpointed that pharmacists should be knowledgeable in terms of PGx interventions since one of their main duties is patients’ education and counselling on their medication [3].

Despite the fact that students were willing to pursue a PGx training in the future, their level of knowledge was moderate and had no significant impact on their intentions to read up with PGx and PM in the future. The PGx and PM knowledge index calculated in this study accrued from the total number of correct answers given to 10 statements already used and validated in other studies [15, 25, 27]. Moreover, the knowledge index, employed, managed to adequately classify participants in terms of their level of PGx and PM knowledge, as the relevant diagram of the overall knowledge assessment yielded, approximately, a normal distribution curve. Surprisingly, most students failed to answer three knowledge questions related to PGx clinical implementation. Indeed, only 20% of respondents knew that PGx clinical guidelines weren’t available for most of the medications, a fact that highlights students’ need for less theoretical sessions and better information about PGx testing availability in a commercial level. Students of the last three academic years demonstrated almost equal level of knowledge in the discipline, but much higher than those of the first and second year, also underpinning the necessity for more PGx-relevant lectures. Pop et al., 2022 made a relevant observation about information gap among Romanian pharmacists [8]. They pointed out that even if Romanian pharmacists had a relatively high level of theoretical PGx knowledge, most of them weren’t aware that PGx testing was commercially available in their country for many medications [8]. Low level of knowledge and lack of information are significant barriers to PGx clinical implementation [10].

The fact that knowledge did not affect students’ intentions for postgraduate training may be attributed, inter alia, to the low pace of PGx and PM implementation in clinical practice. It was found that students’ intentions for postgraduate training in PGx and PM faded approaching to the completion of their studies. In fact, fifth-year students were to some extent, reluctant to attend a relevant MSc course. By the completion of the questionnaire, almost all graduates have attended internships at community and hospital pharmacies and quite probably realised the limited PGx and PM adoption in the clinical setting. Therefore, they likely aspire to postgraduate training in other more promising fields that would facilitate their initial career steps.

The low level of students’ self-confidence coupled with their moderate training satisfaction rate, as well, raise several questions about the efficacy of current pharmacy curriculum. Evidently, school’s curriculum needs a change, especially considering that our study results revealed that both student self-confidence and training satisfaction affected positively their attitudes towards PGx implementation in practice, and consequently, their intentions to read up with PGx and PM. By incorporating case studies, publications, or even journal club sessions dedicated to PM and its clinical interventions to the existing courses, students would better understand the correlation of all modules and gain a deeper knowledge of PM. This could also be improved by the addition of an elective course about the application of PM and PGx in a pharmacist’s clinical practice, to wit, a change from knowledge-based PGx training to a more practical one. This kind of addition would bear fruitful results because pharmacists are more interested in receiving formal training related to drug therapy recommendations, results interpretation, patient counselling and education, future advances as many studies claimed [13, 43, 49].

## Limitations

Our study has a few limitations. The study sample consisted only of undergraduate pharmacy students from the University of Patras, and it didn’t represent all pharmacy schools in Greece. Survey’s questionnaire couldn’t include all factors that potentially affect students’ intentions to pursue postgraduate studies in PGx. Finally, study results may be biased by the COVID-19 pandemic restrictions to teaching processes.

## Conclusion

Pharmacists play a determinant role in clinical PGx but their presence is still limited. Future generations of pharmacists opted to apply new technologies such as PGx in their practice but they considered themselves as not adequately prepared and seek for postgraduate opportunities to broaden their horizons. This phenomenon is global [7, 15, 26, 40, 47]. The low satisfaction level from pharmacy department’s curriculum along with low self-confidence indicates the need for curriculum modifications to tailor students’ needs. Hence, it is urgent for pharmacy schools to update and upgrade their curricula accordingly and introduce more PGx- and PM-related sessions. Study’s questionnaire proved to be a reliable and promising instrument to interpret pharmacy students’ intentions to pursue postgraduate studies in PGx and PM. Given the close collaboration of physicians and pharmacists in the clinical implementation of PGx, this survey instrument would also be suitable for understanding the underlying factors of medical students’ aspirations to further develop their knowledge and skills in PGx and PM.

## Supplementary Information

**Additional file 1: Table S1**. Gender’s impact on students’ answers; **Table S2** Recent PGx/genetic test impact on students’ answers; **Table S3** Chronic medication’s impact on students’ answers; **Table S4** Another BSc degree’s impact on students’ answers; **Table S5** Study year’s impact on students’ answers; **Fig. S1** SEM diagram of the factors influencing students’ intention to pursue postgraduate training in PGx and PM.

**Additional file 2**: Survey Questionnaire.

## Data Availability

Data generated from this survey are available upon request.

## Abbreviations

ADR: Adverse drug reaction
ASHP: American Society of Health System Pharmacists
AVE: Average variance extracted
HTMT: Heterotrait-monotrait ratio of correlations
CFA: Confirmatory factor analysis
CFI: Comparative fit index
CMIN/DF: Chi-square statistics/degree of freedom
CMV: Common method variance
CR: Composite reliability
GLS: Generalised least squares
G2C2: Genetics/Genomics Competency Center
IFI: Incremental fit index
NFI: Normed fit index
PGET: Person genomic education training
PGLP: Pharmacogenomics/genomics literacy framework for pharmacists
PGx: Pharmacogenomics
PM: Personalised medicine
RFI: Relative fit index
RMSEA: Root-mean-square error of approximation
SEM: Structural equation modelling
SRMR: Standardised root-mean-square residual
Std.Dev: Standard deviation
TLI: Tucker–Lewis index
TPB: Theory of planned behaviour
