# Supplementary material for: Pharmacy students’ attitudes and intentions of pursuing postgraduate studies and training in pharmacogenomics and personalised medicine
Source: Hum Genomics. 2023 Mar 23;17:27. doi: 10.1186/s40246-023-00474-8 (PMC10035981; doi:10.1186/s40246-023-00474-8)

**Supplementary Material 1:** *Supplementary Tables and Figures.*

**Supplementary Table 1:** *Gender’s impact on students’ answers.*

|  | **Male** | **Female** | **Mann-Whitney Test** | |
| --- | --- | --- | --- | --- |
|  | **Mean** | **Mean** | **Z** | **Asymp. Sig.** |
| Lectures | 5.02 | 5.22 | -1.297 | 0.195 |
| Books | 4.15 | 4.19 | -0.351 | 0.725 |
| Lab exercises | 4.59 | 4.71 | -0.686 | 0.493 |
| Online educational material | 4.57 | 4.35 | -1.158 | 0.247 |
| Research articles | 3.89 | 3.84 | -0.191 | 0.849 |
| Supplementary material in e-class | 4.94 | 5.41 | -2.315 | 0.021 |
| Optional assignments | 4.00 | 4.26 | -1.100 | 0.271 |
| ***Evaluation of teaching tools*** | 4.45 | 4.57 | -1.116 | 0.265 |
| Confident to explain PGx results to a patient | 3.59 | 3.61 | -0.226 | 0.822 |
| Confident to discuss PGx test results with a physician | 3.86 | 3.74 | -0.648 | 0.517 |
| Confident to recommend a PGx test to a patient | 3.85 | 3.89 | -0.036 | 0.971 |
| Confident to recommend modification of medication based on PGx test | 3.80 | 3.55 | -1.041 | 0.298 |
| Confident to determine therapeutic areas that PGx testing is necessary | 3.63 | 3.48 | -0.689 | 0.491 |
| ***Self-confidence in implementing PGx in clinical practice*** | 3.74 | 3.65 | -0.801 | 0.423 |
| Satisfied with theoretical training | 4.54 | 4.68 | -1.001 | 0.317 |
| Satisfied with lab training | 4.32 | 4.26 | -0.280 | 0.779 |
| Overall satisfied with departments’ curriculum | 4.40 | 4.49 | -0.727 | 0.467 |
| ***Satisfaction with PGx training*** | 4.42 | 4.48 | -0.582 | 0.561 |
| PGx should be an important part of pharmacy students’ curriculum | 5.40 | 5.74 | -2.269 | 0.023 |
| I intend to keep up with future updates in the field of PGx | 4.67 | 5.10 | -2.496 | 0.013 |
| I will include/incorporate PGx testing in patient care | 4.52 | 5.12 | -3.355 | 0.001 |
| I intend to undergo a PGx testing in the future | 4.59 | 4.81 | -1.232 | 0.218 |
| I would recommend PGx testing to patients | 5.23 | 5.39 | -0.589 | 0.556 |
| I would recommend PGx testing to a family member | 5.35 | 5.39 | -0.005 | 0.996 |
| ***Attitudes-Intentions for PGx implementation*** | 4.96 | 5.26 | -2.145 | 0.032 |
| ***Level of knowledge in PGx and PM*** | 5.41 | 5.85 | -1.987 | 0.047 |
| I would like to pursue with postgraduate studies related to PGx and PM | 3.76 | 4.44 | -3.463 | 0.001 |
| I would like to attend certified training or information programs related to PGx and PM | 4.55 | 5.29 | -3.784 | 0.000 |
| ***Intentions for postgraduate training in PGx and PM*** | 4.15 | 4.87 | -3.794 | 0.000 |

**Supplementary Table 2:** *Recent PGx/genetic test impact on students’ answers.*

|  | **Yes** | **No** | **Mann-Whitney Test** | |
| --- | --- | --- | --- | --- |
|  | **Mean** | **Mean** | **Z** | **Asymp. Sig.** |
| Lectures | 4.82 | 5.19 | -1.213 | 0.225 |
| Books | 3.70 | 4.22 | -1.717 | 0.086 |
| Lab exercises | 4.27 | 4.71 | -1.498 | 0.134 |
| Online educational material | 4.88 | 4.38 | -1.946 | 0.052 |
| Research articles | 4.27 | 3.81 | -1.613 | 0.107 |
| Supplementary material in e-class | 5.33 | 5.23 | -0.299 | 0.765 |
| Optional assignments | 4.24 | 4.16 | -0.348 | 0.728 |
| ***Evaluation of teaching tools*** | 4.50 | 4.53 | -0.069 | 0.945 |
| Confident to explain PGx results to a patient | 3.52 | 3.61 | -0.743 | 0.458 |
| Confident to discuss PGx test results with a physician | 4.12 | 3.75 | -0.972 | 0.331 |
| Confident to recommend a PGx test to a patient | 3.91 | 3.87 | -0.044 | 0.965 |
| Confident to recommend modification of medication based on PGx test | 3.94 | 3.60 | -0.872 | 0.383 |
| Confident to determine therapeutic areas that PGx testing is necessary | 3.61 | 3.52 | -0.228 | 0.819 |
| ***Self-confidence in implementing PGx in clinical practice*** | 3.82 | 3.67 | -0.331 | 0.740 |
| Satisfied with theoretical training | 4.39 | 4.65 | -1.220 | 0.222 |
| Satisfied with lab training | 3.88 | 4.32 | -1.642 | 0.101 |
| Overall satisfied with departments’ curriculum | 4.21 | 4.48 | -1.163 | 0.245 |
| ***Satisfaction with PGx training*** | 4.16 | 4.49 | -1.540 | 0.124 |
| PGx should be an important part of pharmacy students’ curriculum | 5.45 | 5.64 | -0.947 | 0.343 |
| I intend to keep up with future updates in the field of PGx | 5.00 | 4.95 | -0.512 | 0.609 |
| I will include/incorporate PGx testing in patient care | 4.94 | 4.90 | -0.520 | 0.603 |
| I intend to undergo a PGx testing in the future | 4.79 | 4.73 | -0.451 | 0.652 |
| I would recommend PGx testing to patients | 5.33 | 5.33 | -0.034 | 0.973 |
| I would recommend PGx testing to a family member | 5.70 | 5.34 | -1.619 | 0.106 |
| ***Attitudes-Intentions for PGx implementation*** | 5.20 | 5.15 | -0.464 | 0.643 |
| ***Level of knowledge in PGx and PM*** | 5.79 | 5.68 | -0.295 | 0.768 |
| I would like to pursue with postgraduate studies related to PGx and PM | 4.42 | 4.18 | -0.898 | 0.369 |
| I would like to attend certified training or information programs related to PGx and PM | 5.03 | 5.03 | -0.381 | 0.703 |
| ***Intentions for postgraduate training in PGx and PM*** | 4.73 | 4.60 | -0.798 | 0.425 |

**Supplementary Table 3:** *Chronic medication’s impact on students’ answers.*

|  | **Yes** | **No** | **Mann-Whitney Test** | |
| --- | --- | --- | --- | --- |
|  | **Mean** | **Mean** | **Z** | **Asymp. Sig.** |
| Lectures | 5.21 | 5.06 | -1.073 | 0.283 |
| Books | 4.17 | 4.18 | -0.043 | 0.966 |
| Lab exercises | 4.59 | 4.79 | -1.084 | 0.278 |
| Online educational material | 4.41 | 4.46 | -0.312 | 0.755 |
| Research articles | 3.92 | 3.76 | -0.865 | 0.387 |
| Supplementary material in e-class | 5.30 | 5.15 | -0.811 | 0.417 |
| Optional assignments | 4.04 | 4.37 | -1.436 | 0.151 |
| ***Evaluation of teaching tools*** | 4.52 | 4.54 | -0.285 | 0.776 |
| Confident to explain PGx results to a patient | 3.57 | 3.65 | -0.455 | 0.649 |
| Confident to discuss PGx test results with a physician | 3.82 | 3.72 | -0.461 | 0.645 |
| Confident to recommend a PGx test to a patient | 3.89 | 3.85 | -0.186 | 0.853 |
| Confident to recommend modification of medication based on PGx test | 3.73 | 3.47 | -1.160 | 0.246 |
| Confident to determine therapeutic areas that PGx testing is necessary | 3.48 | 3.60 | -0.630 | 0.529 |
| ***Self-confidence in implementing PGx in clinical practice*** | 3.70 | 3.66 | -0.261 | 0.794 |
| Satisfied with theoretical training | 4.61 | 4.65 | -0.176 | 0.860 |
| Satisfied with lab training | 4.21 | 4.40 | -0.775 | 0.439 |
| Overall satisfied with departments’ curriculum | 4.44 | 4.49 | -0.217 | 0.828 |
| ***Satisfaction with PGx training*** | 4.42 | 4.51 | -0.420 | 0.675 |
| PGx should be an important part of pharmacy students’ curriculum | 5.63 | 5.60 | -0.182 | 0.856 |
| I intend to keep up with future updates in the field of PGx | 4.99 | 4.89 | -0.471 | 0.638 |
| I will include/incorporate PGx testing in patient care | 4.95 | 4.84 | -0.971 | 0.331 |
| I intend to undergo a PGx testing in the future | 4.81 | 4.60 | -0.881 | 0.378 |
| I would recommend PGx testing to patients | 5.46 | 5.12 | -2.580 | 0.010 |
| I would recommend PGx testing to a family member | 5.53 | 5.11 | -2.779 | 0.005 |
| ***Attitudes-Intentions for PGx implementation*** | 5.23 | 5.03 | -1.761 | 0.078 |
| ***Level of knowledge in PGx and PM*** | 5.88 | 5.38 | -2.805 | 0.005 |
| I would like to pursue with postgraduate studies related to PGx and PM | 4.21 | 4.18 | -0.021 | 0.983 |
| I would like to attend certified training or information programs related to PGx and PM | 5.12 | 4.88 | -1.089 | 0.276 |
| ***Intentions for postgraduate training in PGx and PM*** | 4.66 | 4.53 | -0.486 | 0.627 |

**Supplementary Table 4:** *Another BSc degree’s impact on students’ answers.*

|  | **Yes** | **No** | **Mann-Whitney Test** | |
| --- | --- | --- | --- | --- |
|  | **Mean** | **Mean** | **Z** | **Asymp. Sig.** |
| Lectures | 4.81 | 5.19 | -1.707 | 0.088 |
| Books | 3.91 | 4.20 | -0.998 | 0.318 |
| Lab exercises | 4.03 | 4.73 | -2.500 | 0.012 |
| Online educational material | 4.41 | 4.43 | -0.111 | 0.912 |
| Research articles | 4.16 | 3.83 | -1.020 | 0.308 |
| Supplementary material in e-class | 5.16 | 5.25 | -0.540 | 0.590 |
| Optional assignments | 3.88 | 4.19 | -0.932 | 0.351 |
| ***Evaluation of teaching tools*** | 4.33 | 4.55 | -1.563 | 0.118 |
| Confident to explain PGx results to a patient | 3.22 | 3.64 | -1.262 | 0.207 |
| Confident to discuss PGx test results with a physician | 3.75 | 3.79 | -0.173 | 0.863 |
| Confident to recommend a PGx test to a patient | 3.84 | 3.88 | -0.092 | 0.927 |
| Confident to recommend modification of medication based on PGx test | 3.72 | 3.63 | -0.246 | 0.806 |
| Confident to determine therapeutic areas that PGx testing is necessary | 3.56 | 3.53 | -0.102 | 0.919 |
| ***Self-confidence in implementing PGx in clinical practice*** | 3.62 | 3.69 | -0.167 | 0.867 |
| Satisfied with theoretical training | 4.75 | 4.61 | -0.392 | 0.695 |
| Satisfied with lab training | 4.13 | 4.30 | -0.676 | 0.499 |
| Overall satisfied with departments’ curriculum | 4.50 | 4.45 | -0.086 | 0.931 |
| ***Satisfaction with PGx training*** | 4.46 | 4.45 | -0.299 | 0.765 |
| PGx should be an important part of pharmacy students’ curriculum | 5.03 | 5.68 | -2.732 | 0.006 |
| I intend to keep up with future updates in the field of PGx | 4.41 | 5.01 | -1.925 | 0.054 |
| I will include/incorporate PGx testing in patient care | 4.47 | 4.95 | -1.495 | 0.135 |
| I intend to undergo a PGx testing in the future | 4.13 | 4.79 | -2.312 | 0.021 |
| I would recommend PGx testing to patients | 5.28 | 5.34 | -0.497 | 0.619 |
| I would recommend PGx testing to a family member | 5.50 | 5.36 | -0.377 | 0.706 |
| ***Attitudes-Intentions for PGx implementation*** | 4.80 | 5.19 | -1.994 | 0.046 |
| ***Level of knowledge in PGx and PM*** | 5.94 | 5.67 | -0.545 | 0.586 |
| I would like to pursue with postgraduate studies related to PGx and PM | 3.41 | 4.28 | -2.522 | 0.012 |
| I would like to attend certified training or information programs related to PGx and PM | 4.53 | 5.08 | -1.604 | 0.109 |
| ***Intentions for postgraduate training in PGx and PM*** | 3.97 | 4.68 | -2.090 | 0.037 |

**Supplementary Table 5:** *Study year’s impact on students’ answers.*

|  | **1^st^** | **2^nd^** | **3^rd^** | **4^th^** | **5^th^** | **Kruskal-Wallis Test** | |
| --- | --- | --- | --- | --- | --- | --- | --- |
|  | **Mean** | **Mean** | **Mean** | **Mean** | **Mean** | **Kruskal-Wallis H** | **Asymp. Sig.** |
| Lectures | 4.61 | 5.90 | 5.56 | 4.98 | 4.75 | 43.289 | 0.000 |
| Books | 4.34 | 4.34 | 3.81 | 4.30 | 4.22 | 6.710 | 0.152 |
| Lab exercises | 5.15 | 5.31 | 4.20 | 4.81 | 4.29 | 28.808 | 0.000 |
| Online educational material | 4.56 | 4.41 | 4.77 | 4.40 | 4.06 | 8.734 | 0.068 |
| Research articles | 3.79 | 4.03 | 3.94 | 3.84 | 3.73 | 1.063 | 0.900 |
| Supplementary material in e-class | 5.37 | 5.34 | 5.57 | 4.84 | 4.99 | 13.641 | 0.009 |
| Optional assignments | 3.87 | 5.02 | 4.46 | 4.07 | 3.59 | 25.467 | 0.000 |
| ***Evaluation of teaching tools*** | 4.53 | 4.91 | 4.62 | 4.46 | 4.23 | 16.358 | 0.003 |
| Confident to explain PGx results to a patient | 3.98 | 3.18 | 3.67 | 3.58 | 3.57 | 7.281 | 0.122 |
| Confident to discuss PGx test results with a physician | 4.55 | 3.25 | 3.62 | 3.91 | 3.72 | 18.370 | 0.001 |
| Confident to recommend a PGx test to a patient | 4.32 | 3.54 | 3.74 | 4.07 | 3.82 | 6.811 | 0.146 |
| Confident to recommend modification of medication based on PGx test | 4.26 | 3.16 | 3.49 | 3.74 | 3.61 | 11.358 | 0.023 |
| Confident to determine therapeutic areas that PGx testing is necessary | 4.11 | 3.28 | 3.27 | 3.98 | 3.33 | 15.331 | 0.004 |
| ***Self-confidence in implementing PGx in clinical practice*** | 4.25 | 3.28 | 3.56 | 3.86 | 3.61 | 15.321 | 0.004 |
| Satisfied with theoretical training | 4.50 | 5.36 | 4.77 | 4.58 | 4.14 | 30.240 | 0.000 |
| Satisfied with lab training | 4.84 | 5.23 | 3.74 | 4.05 | 3.90 | 46.470 | 0.000 |
| Overall satisfied with departments’ curriculum | 4.82 | 5.25 | 4.31 | 4.53 | 3.81 | 44.062 | 0.000 |
| ***Satisfaction with PGx training*** | 4.72 | 5.28 | 4.27 | 4.39 | 3.95 | 45.434 | 0.000 |
| PGx should be an important part of pharmacy students’ curriculum | 6.10 | 6.03 | 5.48 | 5.44 | 5.26 | 27.840 | 0.000 |
| I intend to keep up with future updates in the field of PGx | 5.65 | 5.41 | 4.76 | 4.79 | 4.45 | 34.144 | 0.000 |
| I will include/incorporate PGx testing in patient care | 5.45 | 5.41 | 4.95 | 4.49 | 4.39 | 36.863 | 0.000 |
| I intend to undergo a PGx testing in the future | 5.00 | 4.89 | 4.77 | 4.65 | 4.46 | 4.267 | 0.371 |
| I would recommend PGx testing to patients | 5.50 | 5.62 | 5.38 | 5.23 | 5.04 | 9.640 | 0.047 |
| I would recommend PGx testing to a family member | 5.40 | 5.72 | 5.50 | 5.12 | 5.14 | 9.616 | 0.047 |
| ***Attitudes-Intentions for PGx implementation*** | 5.52 | 5.51 | 5.14 | 4.95 | 4.79 | 28.034 | 0.000 |
| ***Level of knowledge in PGx and PM*** | 4.65 | 4.85 | 6.13 | 6.21 | 6.29 | 56.570 | 0.000 |
| I would like to pursue with postgraduate studies related to PGx and PM | 5.16 | 4.77 | 4.30 | 3.74 | 3.33 | 49.674 | 0.000 |
| I would like to attend certified training or information programs related to PGx and PM | 5.50 | 5.52 | 5.23 | 4.70 | 4.38 | 28.486 | 0.000 |
| ***Intentions for postgraduate training in PGx and PM*** | 5.33 | 5.15 | 4.76 | 4.22 | 3.85 | 46.574 | 0.000 |

**Supplementary Figure 1**: *SEM diagram of the factors influencing students’ intention to pursue postgraduate training in PGx and PM.*


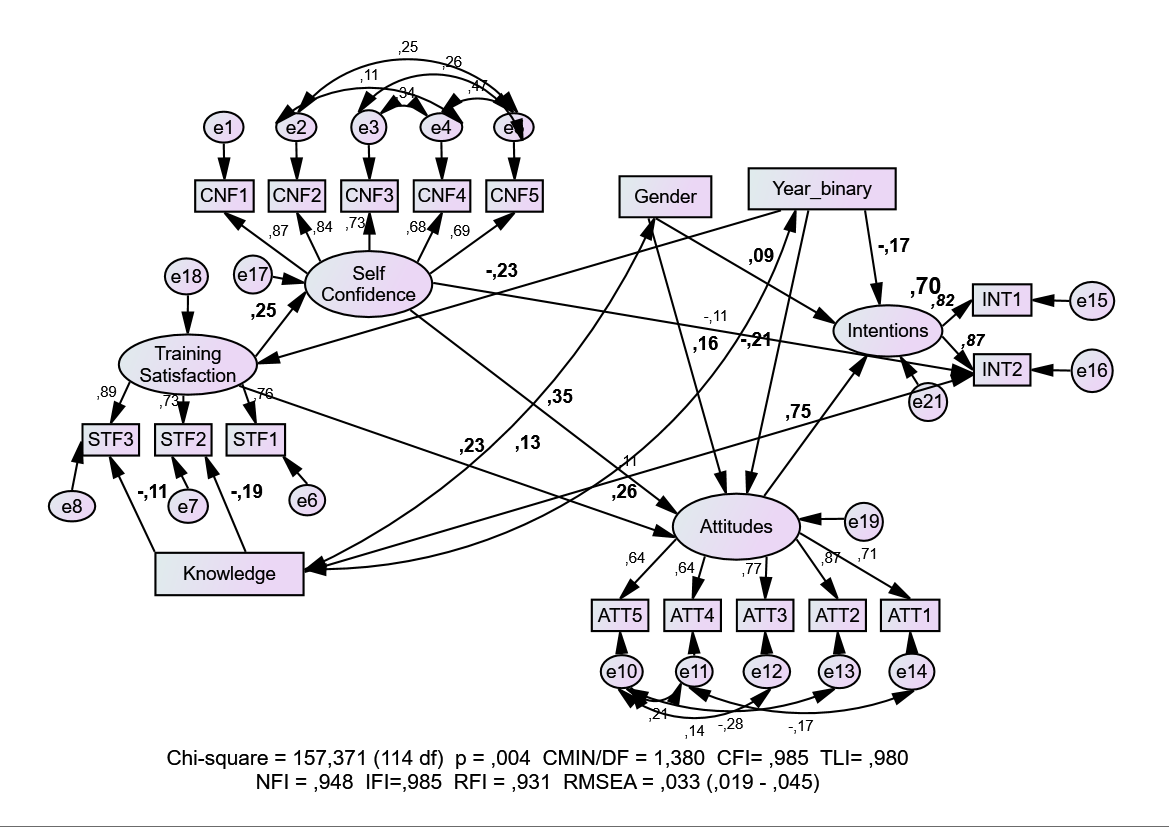

Supplement: Supplementary file 1 — Additional file 1: Table S1. Gender’s impact on students’ answers; Table S2 Recent PGx/genetic test impact on students’ answers; Table S3 Chronic medication’s impact on students’ answers; Table S4 Another BSc degree’s impact on students’ answers; Table S5 Study year’s impact on students’ answers; Fig. S1 SEM diagram of the factors influencing students’ intention to pursue postgraduate training in PGx and PM. [file 40246_2023_474_MOESM1_ESM.docx]
