# Supplementary material for: Pharmacy students’ attitudes and intentions of pursuing postgraduate studies and training in pharmacogenomics and personalised medicine
Source: Hum Genomics. 2023 Mar 23;17:27. doi: 10.1186/s40246-023-00474-8 (PMC10035981; doi:10.1186/s40246-023-00474-8)
Supplement: Supplementary file 2 — Additional file 2: Survey Questionnaire. [file 40246_2023_474_MOESM2_ESM.docx]

**Supplementary Material 2:** *Survey Questionnaire.*

**QUESTIONNAIRE**

This questionnaire was designed as a survey instrument for a research project of the Laboratory of PGx and Individualized Therapy, Department of Pharmacy, University of Patras, investigating health science student’s perceptions about pharmacogenomics and personalised medicine.

The total completion time of this questionnaire is estimated at 10 min. It is highly important to answer all questions. We assure you that your personal data anonymity is preserved. All data collected will be exclusively used for scientific purposes. By completing and submitting this questionnaire, you consent to participate in this study.

Thank you in advance for your cooperation.

**Useful Definitions**

Personalised Medicine: it refers to the application of all data derived from the genetic makeup of each patient to determine the most suitable treatment for him/her in terms of clinical effectiveness, reduction of adverse events, and early diagnosis of diseases based on patient’s genetic prevalence.

Pharmacogenomics (PGx): It refers to the study of how a person’s genetic makeup can affect one’s response to medications.

Pharmacogenetic testing: Pharmacogenetic testing is based on the methods and approaches applied for the determination of most frequent allelic polymorphisms that can affect patient’s response to drug treatment.

**Section Α**

***Evaluation of teaching tools***

Please indicate the level of usefulness of the following teaching tools for your training about the PGx application in clinical practice (*1 = not at all useful, 7= extremely useful*).

|  | **Not at all** |  |  |  |  |  | **Extremely** |
| --- | --- | --- | --- | --- | --- | --- | --- |
| Lectures | **1** | **2** | **3** | **4** | **5** | **6** | **7** |
| Scientific Books | **1** | **2** | **3** | **4** | **5** | **6** | **7** |
| Lab exercises | **1** | **2** | **3** | **4** | **5** | **6** | **7** |
| Online educational material | **1** | **2** | **3** | **4** | **5** | **6** | **7** |
| Research articles in scientific journals | **1** | **2** | **3** | **4** | **5** | **6** | **7** |
| Supplementary material in e-class | **1** | **2** | **3** | **4** | **5** | **6** | **7** |
| Optional assignments | **1** | **2** | **3** | **4** | **5** | **6** | **7** |

**Section Β**

***Self-confidence in implementing PGx in clinical practice***

Please indicate the level of your self-confidence in implementing PGx in clinical practice (1 = not at all to 7= extremely sure)

|  | **Not at all** |  |  |  |  |  | **Extremely** |
| --- | --- | --- | --- | --- | --- | --- | --- |
| How confident do you feel to explain PGx results to a patient? | **1** | **2** | **3** | **4** | **5** | **6** | **7** |
| How confident do you feel to discuss PGx test results with a physician? | **1** | **2** | **3** | **4** | **5** | **6** | **7** |
| How confident do you feel to recommend a PGx test to a patient? | **1** | **2** | **3** | **4** | **5** | **6** | **7** |
| How confident do you feel to recommend a modification in a patient’s drug treatment or dosage according to PGx test results? | **1** | **2** | **3** | **4** | **5** | **6** | **7** |
| How confident do you feel to determine the therapeutic areas (e.g., cardiac, oncology, psychiatric diseases) that PGx testing is necessary? | **1** | **2** | **3** | **4** | **5** | **6** | **7** |

**Section C**

***Satisfaction with the training in PGx implementation in clinical practice.***

Please indicate your level of agreement on each of the following statements. (1 =Totally Disagree to 7= Totally agree)

|  | **Totally Disagree** |  |  | **Neutral** |  |  | **Totally Agree** |
| --- | --- | --- | --- | --- | --- | --- | --- |
| I am satisfied with my theoretical training. | **1** | **2** | **3** | **4** | **5** | **6** | **7** |
| I am satisfied with my lab training | **1** | **2** | **3** | **4** | **5** | **6** | **7** |
| Overall, I am satisfied with my departments’ curriculum in terms of my training in the clinical implementation of PGx. | **1** | **2** | **3** | **4** | **5** | **6** | **7** |

**Section D**

**Attitudes-Intentions for PGx implementation**

Please indicate your level of agreement on each of the following statements. (1 =Totally Disagree to 7= Totally agree)

|  | **Totally Disagree** |  |  | **Neutral** |  |  | **Totally Agree** |
| --- | --- | --- | --- | --- | --- | --- | --- |
| PGx should be an important part of pharmacy students’ curriculum. | **1** | **2** | **3** | **4** | **5** | **6** | **7** |
| I intend to keep up with future updates in the field of PGx. | **1** | **2** | **3** | **4** | **5** | **6** | **7** |
| I will include/incorporate pharmacogenomic testing in patient care. | **1** | **2** | **3** | **4** | **5** | **6** | **7** |
| I intend to undergo a pharmacogenomic testing in the future. | **1** | **2** | **3** | **4** | **5** | **6** | **7** |
| I would recommend pharmacogenomic testing to patients. | **1** | **2** | **3** | **4** | **5** | **6** | **7** |
| I would recommend pharmacogenomic testing to a family member. | **1** | **2** | **3** | **4** | **5** | **6** | **7** |

**Section Ε**

***Intentions for postgraduate training in PGx and PM.***

Please indicate your level of agreement on each of the following statements. (1 =Totally Disagree to 7= Totally agree)

|  | Totally Disagree |  |  | Neutral |  |  | Totally Agree |
| --- | --- | --- | --- | --- | --- | --- | --- |
| I would like to pursue with postgraduate (master, PhD) studies related to PGx and PM. | **1** | **2** | **3** | **4** | **5** | **6** | **7** |
| I would like to attend certified training or information programs related to PGx and PM. | **1** | **2** | **3** | **4** | **5** | **6** | **7** |

**Section F**

***Level of knowledge in PGx and PM***

Please indicate, based on your knowledge, your level of agreement on each of the following statements.

|  | **Agree** | **Disagree** | **Don’t Know** |
| --- | --- | --- | --- |
| Slight differences in a person’s genome can impact on a person’s reaction to a medication. |  |  |  |
| PGx guidelines in clinical practice are available for most of medications. |  |  |  |
| Any genetic variances determining a person’s response to a medication can change throughout a person’s life. |  |  |  |
| PGx can optimize drug dosage and reduce the incidence rate of adverse drug reactions due to wrong medication. |  |  |  |
| Differences in pharmacodynamics and in interaction between drug and molecular target that are observed in population are due to genetic variances. |  |  |  |
| Pharmacokinetic parameters can have a different action among people due to genetic variance. |  |  |  |
| PGx improve the identification of interactions among different pharmaceutical substances. |  |  |  |
| A person’s response to warfarin is affected by genetic variance in ***CYP2C9*** and ***VKORC I*** gene. |  |  |  |
| A patient can have a higher risk to experience an adverse event/ drug toxicity due to inherited genetic variances and drug-drug interactions. |  |  |  |
| The field of PGx will revolutionize healthcare since it will optimize medication prescription, will spark the interest in undergoing a genetic testing for early disease diagnosis and it will decrease health expenditures for pharmaceutical care. |  |  |  |

**Section G**

***Demographics***

*Please tell us about yourself:*

**G1. Gender:**

| Male |  |  | Female |  |  | Prefer not to say |  |
| --- | --- | --- | --- | --- | --- | --- | --- |

**G2. Year of studies:**

| **1^st^** |  | **2^nd^** |  | **3^rd^** |  | **4^th^** |  | **5^th^** |
| --- | --- | --- | --- | --- | --- | --- | --- | --- |
|  |  |  |  |  |  |  |  |  |

**G3. Are you a bachelor degree holder from another department/school?**

| YES |  |  | NO |  |
| --- | --- | --- | --- | --- |

**G4. Have you or a relative of yours undergone any pharmacogenomic testing recently?**

| YES |  |  | NO |  |
| --- | --- | --- | --- | --- |

**G5. Are you or a relative of yours taking any chronic medication?**

| YES |  |  | NO |  |
| --- | --- | --- | --- | --- |

***Thank you for your participation***
